# Disease‐specific health‐related quality of life trajectories up to 15 years after curative treatment for esophageal cancer—a prospective cohort study

**DOI:** 10.1002/cam4.7466

**Published:** 2024-07-04

**Authors:** Zhao Cheng, Asif Johar, Jesper Lagergren, Anna Schandl, Pernilla Lagergren

**Affiliations:** ^1^ Surgical Care Science, Department of Molecular Medicine and Surgery Karolinska Institutet, Karolinska University Hospital Stockholm Sweden; ^2^ Upper Gastrointestinal Surgery, Department of Molecular medicine and Surgery Karolinska Institutet, Karolinska University Hospital Stockholm Sweden; ^3^ School of Cancer and Pharmaceutical Sciences King's College London UK; ^4^ Department of Surgery and Cancer Imperial College London London UK

**Keywords:** esophageal neoplasm, patient‐reported outcome, risk factor

## Abstract

**Background:**

The presence of distinct long‐term disease‐specific HRQL trajectories after curative treatment for esophageal cancer and factors associated with such trajectories are unclear.

**Materials and Methods:**

This population‐based and longitudinal cohort study included 425 esophageal cancer patients who underwent curative treatment, including esophagectomy, in Sweden in 2001–2005 and were followed up until 2020, that is, 15‐year follow‐up. The outcomes were 10 disease‐specific HRQL symptoms, measured by the well‐validated EORTC QLQ‐OES18 questionnaire at 6 months (*n* = 402 patients), and 3 (*n* = 178), 5 (*n* = 141), 10 (*n* = 92), and 15 years (*n* = 52) after treatment. HRQL symptoms were examined for distinct trajectories by growth mixture models. Weighted logistic regression models provided odds ratios (OR) with 95% confidence intervals (95% CI) for nine factors in relation to HRQL trajectories: age, sex, education, proxy baseline HRQL, comorbidity, tumor histology, chemo(radio)therapy, pathological tumor stage, and postoperative complications.

**Results:**

Distinct HRQL trajectories were identified for each of the 10 disease‐specific symptoms. HRQL trajectories with more symptoms tended to persist or alleviate over time, while trajectories with fewer symptoms were more stable. Eating difficulty had three trajectories: persistently less, persistently moderate, and persistently more symptoms. The OR of having a persistently more eating difficulty trajectory was decreased for adenocarcinoma histology (OR = 0.44, 95% CI 0.21–0.95), and increased for pathological tumor stage III‐IV (OR = 2.19, 95% CI 0.99–4.82) and 30‐day postoperative complications (OR = 2.54, 95% CI 1.26–5.12).

**Conclusion:**

Distinct trajectories with long‐term persistent or deteriorating disease‐specific HRQL symptoms were identified after esophageal cancer treatment. Tumor histology, tumor stage, and postoperative complications may facilitate detection of high‐risk patients for unwanted trajectories.

## Graphical abstract

Patients with long‐term persistent or deteriorating disease‐specific health‐related quality of life (HRQL) symptoms following curative treatment for esophageal cancer were identified. Tumor histology, tumor stage, and postoperative complications may facilitate detection of high‐risk patients for unwanted trajectories.

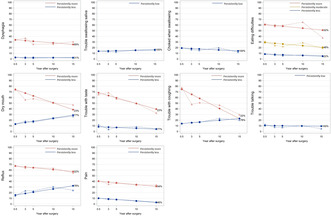

## 1 INTRODUCTION

Esophageal cancer ranks 7th in cancer incidence worldwide and carries a poor prognosis.^1^ Surgery is the backbone of curatively intended treatment, but is usually combined with neoadjuvant therapy for locally advanced esophageal cancer.^2^ Esophagectomy for cancer typically includes removal of the main part of esophagus which is replaced by a gastric tube that is pulled‐up into the chest or the neck and anastomosed to the remaining proximal esophagus.^3^ These major anatomical changes often lead to substantial deterioration in health‐related quality of life (HRQL).

Esophageal cancer‐specific symptoms, for example, dysphagia, dry mouth, and reflux, stand independent of more general HRQL aspects (e.g., physical function) and may persist after surgery, but the long‐term development and whether distinct patterns exist is unknown.^4^, ^5^ Patient, tumor, and treatment characteristics are associated with HRQL aspects measured at certain time points,^6^, ^7^, ^8^, ^9^, ^10^, ^11^ but determinants of distinct longitudinal disease‐specific HRQL trajectories remain to be revealed.

This study aimed to identify possible distinct disease‐specific HRQL symptom trajectories up to 15 years after esophagectomy for esophageal cancer and to reveal factors associated with unwanted trajectories.

## 2 MATERIALS AND METHODS

### 2.1 Study design

This was a Swedish nationwide and prospective cohort study of patients having been curatively treated, including surgical resection (esophagectomy), for esophageal or gastroesophageal junctional cancer (adenocarcinoma or squamous cell carcinoma) between April 2, 2001, and December 31, 2005, with follow‐up until December 31, 2020. The patients were assessed regularly regarding HRQL measurements for up to 15 years after esophagectomy. All participating patients gave written informed consent. The study was approved by the Regional Ethical Review Board in Stockholm, Sweden. The work has been reported in line with the STROCSS criteria.^12^

### 2.2 Data collection

The data collection has been described in detail elsewhere.^13^, ^14^ Briefly, patients were identified from 174 hospital departments involved in the diagnosis or treatment of esophageal cancer and from all seven regional cancer centers in Sweden. Information regarding age, sex, tumor histology, treatment, pathological tumor stage, and 30‐day postoperative complications was collected by review of medical records according to predefined definitions and categorizations to ensure uniformity and completeness. Education data were collected from the Longitudinal Integration Database for Health Insurance and Labour Market (LISA). Data on comorbidity were extracted from the Swedish National Patient Register and were included in the most well‐validated version of the Charlson comorbidity index.^15^ Vital status was checked from the National Register of the Total Population before each follow‐up. HRQL data were collected by questionnaires mailed to the patients at 6 months, 3, 5, 10, and 15 years postoperatively. The study included all patients with at least one HRQL assessment. To obtain proxy baseline (before cancer diagnosis) HRQL information, a random sample of 6969 individuals from the Swedish population was invited, and 4910 (70.5%) completed the same HRQL questionnaires.^16^ Each patient in the study cohort was matched to individuals from the population sample by age, sex, education, and comorbidity. The proxy baseline HRQL was calculated as the mean HRQL scores of the matched population sample.

### 2.3 HRQL outcomes

The study outcomes were disease‐specific HRQL symptom trajectories (categorical) measured by the European Organisation for Research and Treatment of Cancer Quality of Life Oesophageal Cancer Module 18 questionnaire (EORTC QLQ‐OES18).^4^ This is an 18‐item well‐validated questionnaire measuring esophageal cancer‐specific symptoms. It comprises four multi‐item scales (dysphagia, eating difficulties, reflux, and pain) and six single items (trouble swallowing saliva, choking when swallowing, dry mouth, trouble with taste, trouble with coughing, and trouble talking). The response alternatives make up a four‐point Likert scale: [1] “Not at all,” [2] “A little,” [3] “Quite a bit,” and [4] “Very much.” The responses were transformed into a score within the range of 0–100. High scores in scales and single items represent high level of symptoms. Missing data were handled according to the EORTC scoring manual.^17^

### 2.4 Underlying factors

Nine predefined factors that might be associated with disease‐specific HRQL symptom trajectory categories were examined: Age at surgery (continuous variable), sex (female or male), education (<9, 9–12, and >12 years of education), proxy baseline HRQL scores (continuous variable), comorbidity (Charlson comorbidity index score 0, 1 or ≥2, not counting the esophageal cancer), tumor histology (squamous cell carcinoma or adenocarcinoma), chemo(radio)therapy (no or yes), pathological tumor stage (0–I, II, or III–IV), and 30‐day postoperative complications (no or yes, details provided in the Supplementary Methods). These nine factors were selected based on the results of previous research.^5^, ^6^, ^7^, ^8^, ^9^, ^10^, ^11^, ^18^

### 2.5 Statistical analysis

#### 2.5.1 Trajectory analysis

Growth mixture models, a latent‐class analytic approach for identification of homogeneous subgroups within a larger heterogeneous population,^19^, ^20^, ^21^, ^22^ were used to identify distinct disease‐specific HRQL symptom trajectories. A single linear trajectory was first modeled for each HRQL symptom. For each HRQL symptom, up to four trajectories were included into the model depending on HRQL scales and items. We assumed different latent or residual variance and trajectory shape (linear or quadratic) until the best fit indices were reached with successful convergence. Model fit index was calculated using six methods: Akaike Information Criterion (AIC), Bayesian Information Criterion (BIC), sample‐size adjusted BIC, entropy, Vuong‐Lo–Mendell–Rubin test (VLMR), and adjusted Lo–Mendell–Rubin test (aLMR).^23^ AIC, BIC, and sample‐size adjusted BIC compare the log‐likelihood of nested models, where smaller values represent better model fit. Entropy implies the uncertainty of trajectory classification, where higher values indicate higher accuracy. VLMR and aLMR compare the K‐trajectory model with the (K‐1)‐trajectory model, and a significant *P*‐value (<0.05) indicates good fit of a K‐trajectory model. Apart from fit indices, model parsimony, theoretical justification, and interpretability were also considered when selecting the final trajectory model. Models with less than 15% of the patient count in any identified trajectory were rejected to avoid unstable estimates. The probability of being categorized into a trajectory was derived from the final model for all HRQL symptoms. Patients were assigned to the trajectory category with the highest estimated probability belonging. The model‐estimated mean and sample mean calculated from patient's HRQL score for each trajectory were presented from the growth mixture models. The sample mean was calculated as the mean score of each HRQL symptom of patients assigned into each trajectory with their probability of trajectory category as weights. All available QLQ‐OES18 data were used in the growth mixture models, assuming that data were missing at random.

#### 2.5.2 Analysis of associations between underlying factors and trajectories

Weighted binomial and multinomial logistic regression models were used to calculate odds ratios (OR) with 95% confidence intervals (CI) for associations between the nine underlying factors and HRQL trajectory category.^21^ The reference HRQL trajectory was the trajectory representing the least symptoms. The other studied underlying factors were included in the multivariable models to account for potential confounding. The weights used in the logistic models were the trajectory category probabilities from the growth mixture models. Statistical significance was set at a two‐sided 5% level. SAS version 9.4 software (Cary, North Carolina: SAS Institute Inc.) was used for the data management and analyses, except for the growth mixture model analyses that were done using MPlus version 8.7 software (Los Angeles, California: Muthén & Muthén).

## 3 RESULTS

### 3.1 Patients

**FIGURE 1:**
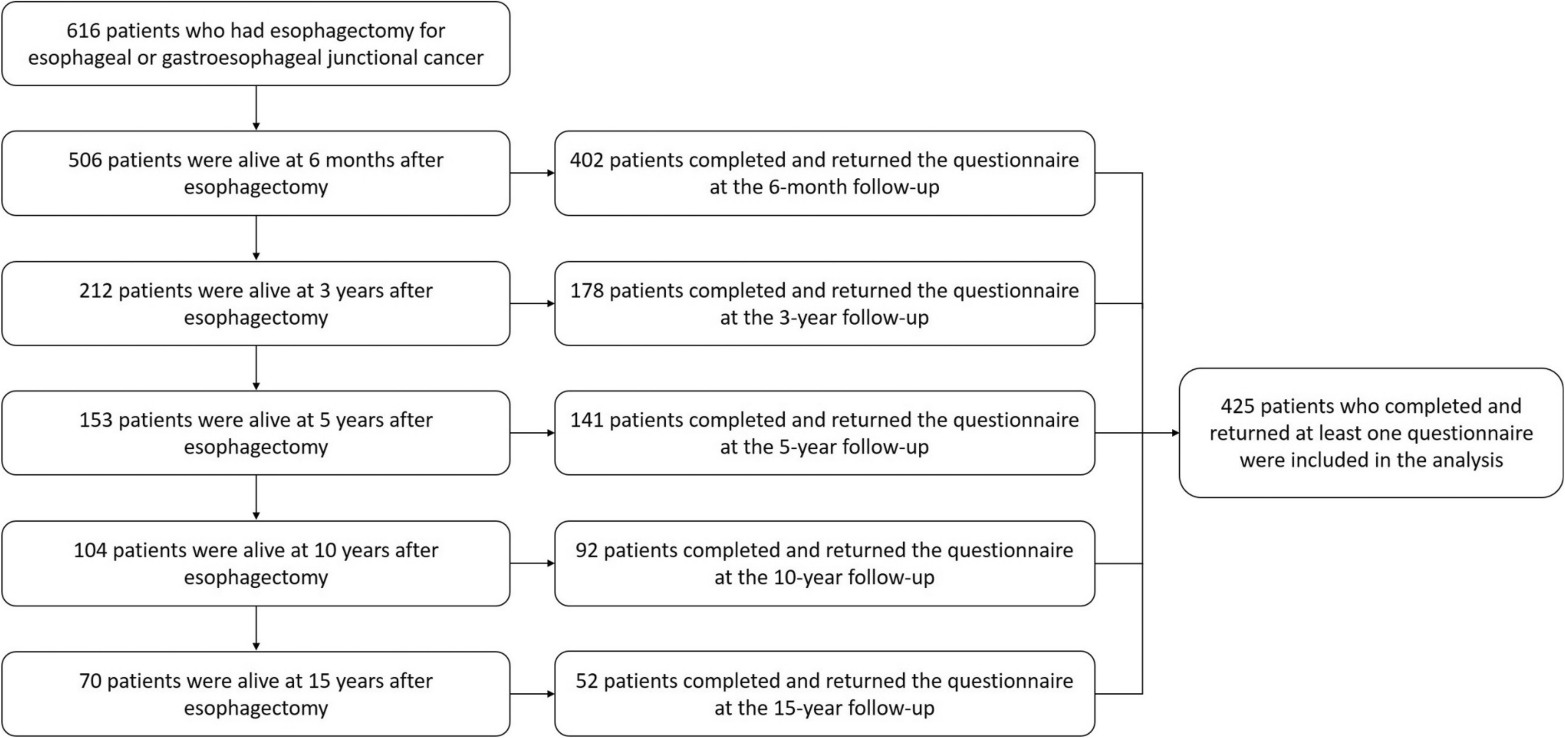
Study flow chart.

The cohort included 616 patients who underwent esophagectomy for esophageal cancer, representing 90% of all eligible patients during the recruitment period. Among the 506 patients who were alive 6 months after the surgery, 402 (79%) responded to the disease‐specific HRQL questionnaire at the 6‐month follow‐up, and 178 out of 212 living patients (84%) responded at 3 years, 141 out of 153 (92%) at 5 years, 92 out of 104 (88%) at 10 years, and 52 out of 70 patients (74%) responded to the questionnaires at 15 years after surgery (Figure 1). The final study cohort included 425 patients who completed and returned at least one of the HRQL questionnaires during the follow‐ups. Characteristics of these participants are presented in Table S1.

### 3.2 Presence of disease‐specific HRQL symptom trajectories

**FIGURE 2:**
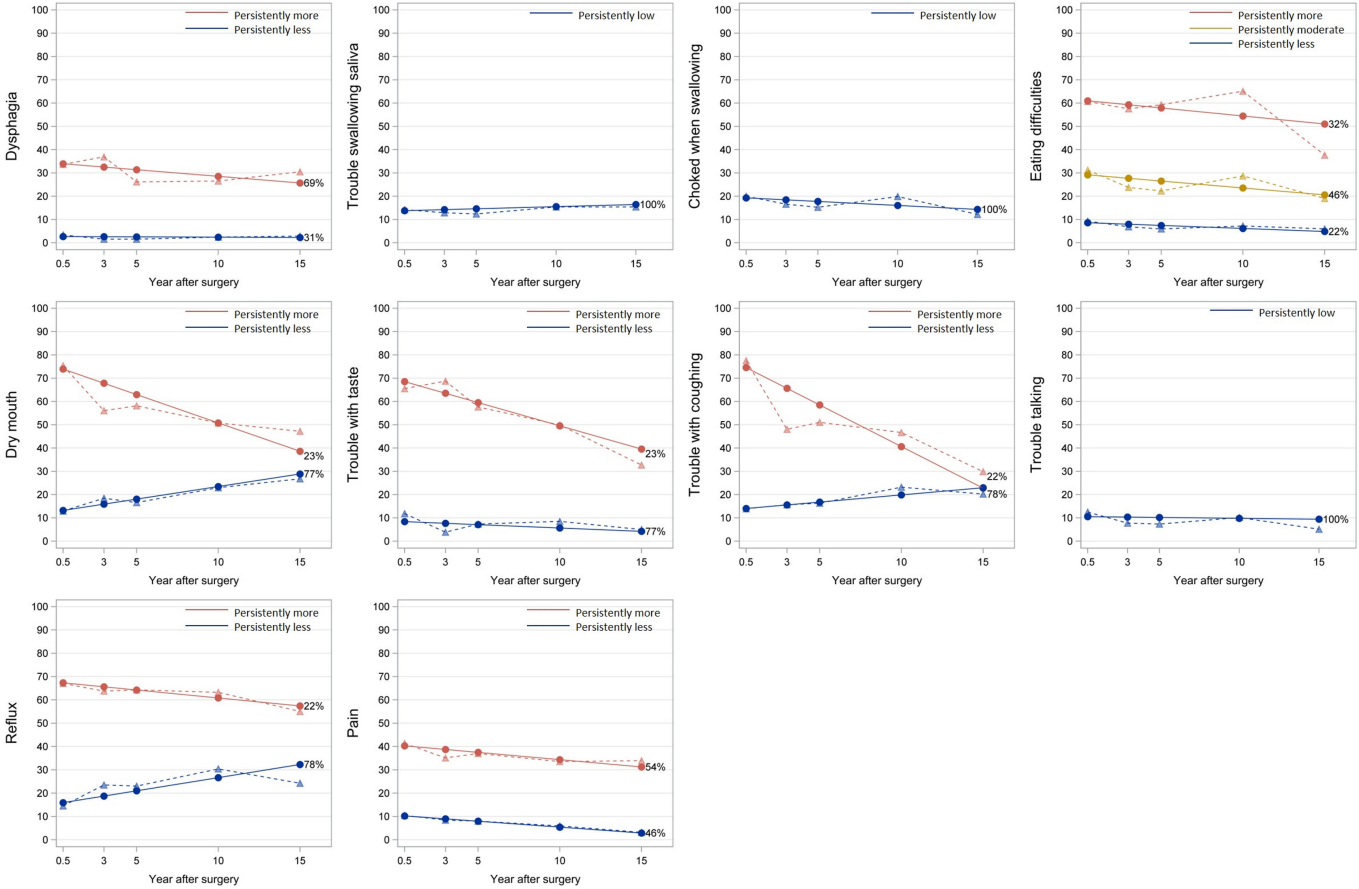
Esophageal cancer specific health‐related quality of life trajectories after esophagectomy. Note: A high symptom score represents a high level of symptom. Solid lines represent model‐estimated mean, and dash lines represent sample‐observed mean. The percentage after each trajectory is the final patient proportion for the trajectory category based on estimated posterior probabilities.

Between 1 and 3 distinct trajectories were identified for each of the 10 measured disease‐specific symptoms (Figure 2). Two trajectories were identified for dysphagia, dry mouth, trouble with taste, reflux, and pain, one representing less symptoms and the other representing more symptoms. Three trajectories were identified for eating difficulties, namely persistently less, persistently moderate, and persistently more symptoms. One trajectory was identified for trouble swallowing saliva, choking when swallowing and trouble talking, representing persistently low symptoms. Fit statistics for model selection are presented in Table S2.

For patients with more troublesome symptoms (red or yellow trajectories in Figure 2), the trajectories tended to either persist or alleviate during follow‐up, but not increase. For patients with less troublesome symptoms (the blue trajectories in Figure 2), most symptoms persisted, except for dry mouth and reflux which worsened over time. Characteristics of patients grouped by the three distinct trajectories of eating difficulties are presented in Table 1. Higher proportions of squamous cell carcinoma histology, advanced pathological tumor stage, and postoperative complications were found among patients with the trajectory of more eating difficulties.

**TABLE 1:** Characteristics of 425 patients who had at least one measurement of eating difficulties after surgery for esophageal cancer.

	Eating difficulty trajectory			
	Persistently less	Persistently moderate	Persistently more	*p*
	Number (%)	Number (%)	Number (%)	
Age				
Mean (standard deviation)	65.5 (9.9)	65.9 (9.3)	65.3 (10.0)	0.840
Sex				
Female	17 (16.8)	36 (17.2)	26 (22.6)	0.429
Male	84 (83.2)	173 (82.8)	89 (77.4)	
Education (years)				
<9	41 (40.6)	97 (46.4)	54 (47.0)	0.742
9–12	42 (41.6)	77 (36.8)	43 (37.4)	
>12	17 (16.8)	31 (14.8)	14 (12.2)	
Missing	1 (1.0)	4 (1.9)	4 (3.5)	
Proxy baseline score of eating difficulties				
Mean (standard deviation)	2.1 (1.7)	2.5 (2.9)	2.4 (2.1)	0.330
Charlson comorbidity index				
0	56 (55.4)	122 (58.4)	66 (57.4)	0.992
1	27 (26.7)	51 (24.4)	29 (25.2)	
≥2	18 (17.8)	36 (17.2)	20 (17.4)	
Tumor histology				
Squamous cell carcinoma	16 (15.8)	47 (22.5)	38 (33.0)	0.010
Adenocarcinoma	85 (84.2)	162 (77.5)	77 (67.0)	
Chemo(radio)therapy				
No	96 (95.0)	197 (94.3)	106 (92.2)	0.646
Yes	5 (5.0)	12 (5.7)	9 (7.8)	
Pathological tumor stage				
0‐I	29 (28.7)	47 (22.5)	21 (18.3)	0.503
II	25 (24.8)	68 (32.5)	33 (28.7)	
III–IV	46 (45.5)	92 (44.0)	59 (51.3)	
Missing	1 (1.0)	2 (1.0)	2 (1.7)	
Postoperative complications				
No	75 (74.3)	134 (64.1)	67 (58.3)	0.046
Yes	26 (25.7)	75 (35.9)	48 (41.7)	

### 3.3 Factors associated with disease‐specific HRQL symptom trajectories

Longer education (>12 years versus <9 years) was associated with lower odds of persistent and troublesome symptoms (red trajectories in Figure 2) regarding dry mouth (OR 0.40, 95% CI 0.17–0.92) and reflux (OR 0.27, 95% CI 0.10–0.78) (Table 2). Compared to squamous cell carcinoma, patients with adenocarcinoma had reduced odds of long‐term eating difficulties (OR 0.44, 95% CI 0.21–0.95) and trouble with coughing (OR 0.51, 95% CI 0.29–0.90). More advanced pathological tumor stage (III‐IV) was associated with increased odds of long‐term eating difficulties (OR 2.19, 95% CI 0.99–4.82) and trouble with taste (OR 2.36, 95% CI 1.19–4.68) compared to earlier stages (0‐I). Patients with postoperative complications had higher odds of long‐term eating difficulties (OR 2.54, 95% CI 1.26–5.12), trouble with taste (OR 1.89, 95% CI 1.13–3.19), trouble with coughing (OR 2.62, 95% CI 1.56–4.41), and pain (OR 1.84, 95% CI 1.16–2.90), compared to those without complications. No statistically significant associations were found for age, sex, proxy baseline HRQL score, comorbidity, or chemo(radio)therapy in relation to any of the disease‐specific HRQL symptom trajectories (Table 2).

**TABLE 2:** Odds ratios (95% confidence intervals) between factors and esophageal cancer specific health‐related quality of life (HRQL) symptom trajectories^a^ after esophagectomy.

	Dysphagia	Eating difficulties		Dry mouth	Trouble with taste	Trouble with coughing	Reflux	Pain
	Persistently less (Reference)^b^	Persistently less (Reference)^b^		Less and deteriorating (Reference)^b^	Persistently less (Reference)^b^	Persistently less (Reference)^b^	Less & deteriorating (Reference)^b^	Persistently less (Reference)^b^
	Persistently more	Persistently moderate	Persistently more	More and alleviating	More and alleviating	More and alleviating	Persistently more	Persistently more
Age								
Continuous	1.00 (0.98–1.02)	1.01 (0.98–1.04)	1.01 (0.97–1.04)	1.02 (0.99–1.05)	1.03 (1.00–1.06)	1.00 (0.97–1.03)	1.01 (0.98–1.04)	0.99 (0.96–1.01)
Sex								
Female	1.00 (Reference)	1.00 (Reference)	1.00 (Reference)	1.00 (Reference)	1.00 (Reference)	1.00 (Reference)	1.00 (Reference)	1.00 (Reference)
Male	1.04 (0.59–1.85)	1.04 (0.48–2.23)	0.80 (0.35–1.84)	0.87 (0.46–1.66)	0.80 (0.42–1.53)	1.57 (0.76–3.24)	0.58 (0.31–1.11)	1.09 (0.61–1.94)
Education (years)								
<9	1.00 (Reference)	1.00 (Reference)	1.00 (Reference)	1.00 (Reference)	1.00 (Reference)	1.00 (Reference)	1.00 (Reference)	1.00 (Reference)
9–12	**0.63 (0.39–1.02)**	0.94 (0.50–1.77)	0.92 (0.45–1.87)	**0.55 (0.32–0.94)**	**0.57 (0.32–1.01)**	1.04 (0.59–1.84)	0.62 (0.35–1.10)	0.70 (0.44–1.13)
>12	0.98 (0.50–1.92)	1.02 (0.44–2.35)	0.84 (0.32–2.20)	**0.40 (0.17–0.92)**	0.91 (0.43–1.94)	1.04 (0.48–2.28)	**0.27 (0.10–0.78)**	0.59 (0.30–1.13)
Proxy baseline HRQL score^c^								
Continuous	1.16 (0.89–1.53)	1.12 (0.96–1.31)	1.08 (0.90–1.28)	1.01 (0.98–1.05)	1.04 (0.90–1.20)	0.99 (0.96–1.03)	1.00 (0.94–1.06)	1.04 (0.98–1.11)
Charlson comorbidity index								
0	1.00 (Reference)	1.00 (Reference)	1.00 (Reference)	1.00 (Reference)	1.00 (Reference)	1.00 (Reference)	1.00 (Reference)	1.00 (Reference)
1	1.03 (0.60–1.76)	0.69 (0.35–1.36)	0.72 (0.34–1.55)	0.66 (0.35–1.24)	0.87 (0.47–1.61)	0.70 (0.36–1.38)	0.86 (0.44–1.66)	0.66 (0.39–1.11)
≥2	1.55 (0.82–2.92)	0.77 (0.35–1.70)	0.79 (0.33–1.92)	0.79 (0.41–1.54)	0.73 (0.36–1.48)	1.26 (0.64–2.47)	1.05 (0.52–2.13)	0.77 (0.42–1.40)
Tumor histology								
Squamous cell carcinoma	1.00 (Reference)	1.00 (Reference)	1.00 (Reference)	1.00 (Reference)	1.00 (Reference)	1.00 (Reference)	1.00 (Reference)	1.00 (Reference)
Adenocarcinoma	0.73 (0.42–1.24)	0.75 (0.36–1.56)	**0.44 (0.21–0.95)**	0.71 (0.41–1.22)	0.71 (0.40–1.27)	**0.51 (0.29–0.90)**	0.78 (0.43–1.43)	0.84 (0.50–1.41)
Chemo(radio)therapy								
No	1.00 (Reference)	1.00 (Reference)	1.00 (Reference)	1.00 (Reference)	1.00 (Reference)	1.00 (Reference)	1.00 (Reference)	1.00 (Reference)
Yes	1.13 (0.44–2.94)	1.20 (0.31–4.69)	2.11 (0.52–8.48)	1.46 (0.55–3.83)	1.02 (0.34–3.08)	1.67 (0.63–4.43)	0.77 (0.24–2.46)	1.28 (0.50–3.30)
Pathological tumor stage								
0–I	1.00 (Reference)	1.00 (Reference)	1.00 (Reference)	1.00 (Reference)	1.00 (Reference)	1.00 (Reference)	1.00 (Reference)	1.00 (Reference)
II	1.13 (0.62–2.06)	1.79 (0.85–3.77)	1.97 (0.82–4.76)	0.98 (0.50–1.90)	1.36 (0.63–2.94)	0.89 (0.42–1.89)	1.19 (0.58–2.42)	**1.89 (1.05–3.40)**
III–IV	1.15 (0.67–1.98)	1.15 (0.58–2.26)	**2.19 (0.99–4.82)**	0.85 (0.45–1.58)	**2.36 (1.19–4.68)**	1.38 (0.72–2.66)	0.79 (0.40–1.57)	1.28 (0.75–2.20)
Postoperative complications								
No	1.00 (Reference)	1.00 (Reference)	1.00 (Reference)	1.00 (Reference)	1.00 (Reference)	1.00 (Reference)	1.00 (Reference)	1.00 (Reference)
Yes	1.17 (0.73–1.88)	**2.07 (1.09–3.94)**	**2.54 (1.26–5.12)**	1.31 (0.79–2.17)	**1.89 (1.13–3.19)**	**2.62 (1.56–4.41)**	1.31 (0.75–2.27)	**1.84 (1.16–2.90)**

*Note*: Bold results are (borderline) statistically significant.

^a^ “Trouble swallowing saliva”, “Choked when swallowing,” and “Trouble talking” are not included in the analysis since only one trajectory was identified.

^b^ The reference trajectory of each symptom is the corresponding blue trajectory with less symptom in Figure 1.

^c^ The proxy baseline HRQL scores are the corresponding baseline scores of each symptom.

## 4 DISCUSSION

This study revealed distinctly different symptom trajectories up to 15 years after treatment for esophageal cancer. Most trajectories differed by being persistently stable, more troublesome symptoms but improving, and less troublesome symptoms but deteriorating. The numbers and patterns of trajectories differed for the specific symptoms analyzed. Shorter education, squamous cell carcinoma histology, advanced pathological tumor stage, and postoperative complications were associated with increased risks of trajectories with more troublesome symptoms.

Strengths of the study include the nationwide and population‐based cohort design with collection of data from medical records and high‐quality registers, the longitudinal assessment of HRQL data for up to 15 years after treatment from a well‐validated questionnaire, the high response rates, and the assessment of a proxy HRQL baseline. These strengths indicate validity and generalizability of the findings. There are also weaknesses. The poor survival reduced the number of study participants and thus the statistical power. To still achieve reliable estimates, at least 15% of the participants were required in each trajectory, but this approach reduced the ability to identify trajectories of small patient groups. Treatment practices changed during the study period, for example, increased use of neoadjuvant chemo(radio)therapy and minimally invasive surgery. However, the HRQL was similar in patients with and without neoadjuvant chemo(radio)therapy^24^, ^25^ or minimally invasive versus open surgery,^26^ indicating that these changes did not much influence the results. Unmeasured or residual confounding are inevitable in observational studies, and factors potentially associated with esophageal cancer‐specific symptoms, such as physical activity, might have influenced the associations between underlying factors and HRQL trajectories. However, the results were adjusted for several factors, which should reduce confounding.

To the best of our knowledge, this is the first study to identify distinct long‐term trajectories of esophageal cancer‐specific symptoms.^4^ Three symptoms imply the existence of treatment‐related side effects, that is, dry mouth, trouble with coughing, and trouble talking.^27^ About 20% of the patients had severe problems of dry mouth and coughing 6 months after surgery, but these symptoms gradually relieved over time. Patients might have trouble talking and voice changes after several hours of surgery, possibly due to bruises of the vocal cords, regurgitation, and swelling of tissues in the neck. But according to the current results, these problems had negligible influence after the initial 6‐month assessment. Only 1 trajectory with persistent less symptoms related to swallowing, that is, trouble swallowing saliva and choking when swallowing, was identified, indicating that swallowing problems may not be major long‐term concerns after treatment. Regarding eating problems, that is, eating difficulties and trouble with taste, 3 and 2 distinct trajectories were identified, respectively. Eating difficulties included troubles enjoying meals, troublesome eating, concerns with eating in front of others and feeling full too quickly.^4^ Patients with eating difficulties at the 6‐month assessment seemed to have this problem persistently also in the long term, while trouble with taste decreased over time. This study found that as many as about 70% of the patients had persistently high levels of dysphagia. Though dysphagia could occur secondary to anastomosis scarring and fibrosis after surgery,^28^ the high rate might also be partly due to that questions about dysphagia in the questionnaire concerned eating ability rather than swallowing difficulties: “Could you eat food or drink liquids?” rather than “Have you had trouble swallowing food or drinking liquids?”. Reflux and regurgitation are common after esophagectomy because the lower gastroesophageal sphincter is removed, and the acidity of gastric contents is gradually regained because of vagal reinnervation.^28^, ^29^ This is supported by the study findings showing one trajectory with persistently more troublesome reflux, and another trajectory with initially less but increasing regurgitation problems. The multi‐item pain scale in the QLQ‐OES18 questionnaire includes eating, chest and stomach pain. Half of the patients reported a persistently increased level of pain, while others had almost no such pain throughout the follow‐up.

Some specific underlying factors, that is, education, tumor histology and pathological tumor stage, and postoperative complications, were associated with trajectories of esophageal cancer‐specific symptoms. Compared to patients with short education and a cancer of squamous cell carcinoma histology, those with longer education and adenocarcinoma histology may have healthier lifestyle habits, for example, less smoking and alcohol consumption, which could contribute to less HRQL symptoms. Patients with advanced tumor stage might experience more psychological burden, possibly increasing the risk of remaining symptoms. Postoperative complications are known risk factors for poor HRQL and are specifically associated with pain, fatigue, and delayed recovery after esophagectomy.^30^, ^31^, ^32^

## 5 CONCLUSION

This study describes a comprehensive picture of disease‐specific HRQL development for up to 15 years after curative treatment for esophageal cancer. The results highlight the importance of understanding how specific symptoms change over time after treatment. Patients with shorter education, squamous cell carcinoma histology, advanced pathological tumor stage, and postoperative complications seem to be more prone to have persistent or worsening symptoms and might thus require a closer follow‐up with tailored interventions.

## AUTHOR CONTRIBUTIONS

**Zhao Cheng:** Conceptualization (equal); formal analysis (equal); methodology (equal); software (equal); visualization (equal); writing – original draft (equal); writing – review and editing (equal). **Asif Johar:** Data curation (equal); formal analysis (equal); methodology (equal); software (equal); writing – review and editing (equal). **Jesper Lagergren:** Resources (equal); writing – review and editing (equal). **Anna Schandl:** Writing – review and editing (equal). **Pernilla Lagergren:** Conceptualization (equal); funding acquisition (equal); project administration (equal); resources (equal); supervision (equal); writing – review and editing (equal).

## FUNDING INFORMATION

This study is funded by the Swedish Cancer Society, the Swedish Research Council, and the Cancer Research Funds of Radiumhemmet. Pernilla Lagergren is supported by NIHR Imperial Biomedical Research Centre (BRC) for her Imperial College London affiliation.

## CONFLICT OF INTEREST STATEMENT

The authors declare no conflicts of interest.

## ETHICS APPROVAL STATEMENT

The study was approved by the Regional Ethical Review Board in Stockholm, Sweden (diary number 2015/0091–32).

## PATIENT CONSENT STATEMENT

All participating patients gave written informed consent.

## Supporting information


Data S1.

## Data Availability

The data of this study are not publicly available due to ethical restrictions. The data are available from the corresponding author P.L. on reasonable request.
