# Supplementary material for: Disease‐specific health‐related quality of life trajectories up to 15 years after curative treatment for esophageal cancer—a prospective cohort study
Source: Cancer Med. 2024 Jul 4;13(13):e7466. doi: 10.1002/cam4.7466 (PMC11222968; doi:10.1002/cam4.7466)
Supplement: Supplementary file 1 — Data S1. [file CAM4-13-e7466-s001.docx]

**Title:** **Disease-specific health-related quality of life trajectories up to 15 years after** **curative treatment for esophageal cancer - a prospective cohort study**

**Supplementary Materials - Index**

| **Supplementary eMethods** |  |
| --- | --- |
| The definition of postoperative complications | *pag. 2* |
| **Supplementary Results** |  |
| eTable 1. Characteristics of 425 patients who had at least one measurement of health-related quality of life (HRQL) after surgery for esophageal cancer | *pag. 3* |
| eTable 2. Fit statistics for model comparison of EORTC QLQ-OES18a symptom scales and items | *pag. 4* |
|  |  |
|  |  |

**Supplementary eMethods**

**The definition of postoperative complications:**

Postoperative complications (no or yes) were defined as deviations from the normal postoperative course within 30 days of surgery. The included complications (with definitions) were: postoperative bleeding (>2000 mL or requiring reoperation), anastomotic insufficiency (clinically significant or radiologically detected), substitute necrosis (clinically significant ischemia with ulceration or perforation), intra-abdominal abscess (≥3*3cm radiologically or surgically detected abscess with clinical symptoms such as fever or pain), intrathoracic abscess or empyema (≥3*3cm radiologically or surgically detected abscess with clinical symptoms such as fever, pain or dyspnea); sepsis (causing clinical symptoms such as fever, chills and proven bacteria in the blood), wound infection (causing clinical symptoms and requiring treatment), wound dehiscence (clinically obvious wound rupture), renal failure (dialysis needed), respiratory insufficiency (reintubation or mechanical ventilation needed), hepatic insufficiency (progressive jaundice), recurrent laryngeal nerve paralysis (laryngeal inspection ascertained), pneumonia (radiologically detected infiltrate with clinical symptoms such as fever, cough or dyspnea), pulmonary embolism (radiologically detected), other embolism (radiologically detected and requiring treatment, deep venous thrombosis (radiologically or clinically verified with treatment needs), ileus (radiologically detected ileus in need of surgery), thoracic ductus injury (thoracic lymph leakage requiring drainage for more than 7 days or reoperation), myocardial infarction (electrocardiogram or cardiac enzymes verified), atrial fibrillation (newly electrocardiogram detected and treatment required), cerebral infarction (radiologically verified), strictures in anastomosis (endoscopic intervention required), gastric perforation (surgical intervention required), and other complications.

**Supplementary Results**

| **eTable 1.**  **Characteristics of 425 patients who had at least one measurement of health-related quality of life (HRQL) after surgery for esophageal cancer** | | |
| --- | --- | --- |
|  | Number | % |
| **Age** |  |  |
| Mean (standard deviation) | 65.6 (9.6) | - |
| **Sex** |  |  |
| Female | 79 | 18.6 |
| Male | 346 | 81.4 |
| **Education (years)** |  |  |
| <9 | 192 | 45.2 |
| 9-12 | 162 | 38.1 |
| >12 | 62 | 14.6 |
| Missing | 9 | 2.1 |
| **Proxy baseline HRQL summary score** | |  |
| Mean (standard deviation) | 2.4 (2.5) | - |
| **Charlson comorbidity index** |  |  |
| 0 | 244 | 57.4 |
| 1 | 107 | 25.2 |
| ≥2 | 74 | 17.4 |
| **Tumour histology** |  |  |
| Squamous cell carcinoma | 101 | 23.8 |
| Adenocarcinoma | 324 | 76.2 |
| **Chemo(radio)therapy** |  |  |
| No | 399 | 93.9 |
| Yes | 26 | 6.1 |
| **Pathological tumour stage** |  |  |
| 0-I | 97 | 22.8 |
| II | 126 | 29.6 |
| III-IV | 197 | 46.4 |
| Missing | 5 | 1.2 |
| **Postoperative complications** |  |  |
| No | 276 | 64.9 |
| Yes | 149 | 35.1 |
|  | | |

| **eTable 2. Fit statistics for model comparison of EORTC QLQ-OES18^a^ symptom scales and items** | | | | | | | | | | | |
| --- | --- | --- | --- | --- | --- | --- | --- | --- | --- | --- | --- |
| **Health-related quality of life** | **Trajectory number** | **Latent variance** | **Latent mean** | **Residual variance** | **AIC^b^** | **BIC^c^** | **Adjusted BIC^d^** | **Entropy** | **P for VLMR^e^** | **P for aLMR^f^** | **Note** |
| **Dysphagia** | 1 | i s | free | free | 8055.115 | 8095.706 | 8063.972 | 0 |  |  |  |
|  | 1 | i s | free | Same within class | 8074.586 | 8098.941 | 8079.9 | 0 |  |  |  |
|  | 2 | i s | Free | Free | 7950.761 | 8003.529 | 7962.275 | 0.903049 | 0 | 0 | 10.8% sample in one trajectory |
|  | **2** | **i s** | **Free** | **Same within class** | **7671.19** | **7711.781** | **7680.047** | **0.755817** | **0.0338** | **0.0374** |  |
|  | 3 | i s | Free | Free | 7909.623 | 7974.569 | 7923.795 | 0.73212 | 0.0076 | 0.0092 | 9.2% sample in one trajectory |
|  | 3 | i s | Free | Same within class | |  |  |  |  |  | Model not terminated normally |
| **Trouble swallowing saliva** | 1 | i s@0 | Free | Free | 7969.598 | 8002.089 | 7976.702 | 0 |  |  |  |
|  | **1** | **i s** | **Free** | **Same within class** | **7969.543** | **7993.911** | **7974.871** | **0** |  |  |  |
|  | 2 | i@0 s | Free | Free | 7803.102 | 7847.778 | 7812.871 | 0.969634 | 0.0001 | 0.0001 | 10.0% sample in one trajectory |
|  | 2 | i s | Free | Same within class | |  |  |  |  |  | Model not terminated normally |
| **Choked when swallowing** | **1** | **i s** | **Free** | **Free** | **7783.632** | **7824.082** | **7792.349** | **0** |  |  |  |
|  | 1 | i s | Free | Same within class | 7793.743 | 7818.013 | 7798.973 | 0 |  |  |  |
|  | 2 | i@0 s | Free | Free | 7727.266 | 7771.761 | 7736.854 | 0.769934 | 0.0462 | 0.0536 |  |
|  | 2 | i s | Free | Same within class | |  |  |  |  |  | Model not terminated normally |
| **Eating difficulties** | 1 | i s | Free | Free | 7770.174 | 7810.695 | 7778.962 | 0 |  |  |  |
|  | 1 | i s | Free | Same within class | 7769.387 | 7793.699 | 7774.659 | 0 |  |  |  |
|  | 2 | i s | Free | Free | 7727.461 | 7780.138 | 7738.884 | 0.693119 | 0.0008 | 0.0012 |  |
|  | 2 | i s | Free | Same within class | 7628.18 | 7668.701 | 7636.968 | 0.689953 | 0 | 0 |  |
|  | 3 | i s | Free | Free | 7710.695 | 7775.528 | 7724.754 | 0.755283 | 0.0038 | 0.0048 | 3.0% sample in one trajectory |
|  | **3** | **i s** | **Free** | **Same within class** | **7562.097** | **7618.826** | **7574.399** | **0.590537** | **0.0002** | **0.0003** |  |
|  | 4 | i s | Free | Free | 7691.092 | 7768.082 | 7707.788 | 0.74229 | 0.158 | 0.1687 | 2.9% sample in one trajectory |
|  | 4 | i s | Free | Same within class | 7549.922 | 7614.756 | 7563.982 | 0.604927 | 0.0404 | 0.0451 | 10.8% sample in one trajectory |
| **Dry mouth** | 1 | i s | Free | Free | 8154.768 | 8195.335 | 8163.602 | 0 |  |  |  |
|  | 1 | i s@0 q | Free | Free | 8152.853 | 8197.477 | 8162.57 | 0 |  |  |  |
|  | 1 | i s | Free | Same within class | 8149.522 | 8173.863 | 8154.823 | 0 |  |  |  |
|  | **2** | **i s** | **Free** | **Free** | **8068.806** | **8121.544** | **8080.29** | **0.871977** | **0** | **0** |  |
|  | 2 | i s | Free | Same within class | |  |  |  |  |  | Model not terminated normally |
|  | 3 | i s | Free | Free | 7899.507 | 7964.416 | 7913.641 | 0.955091 | 0.047 | 0.0537 |  |
|  | 3 | i s | Free | Same within class | |  |  |  |  |  | Model not terminated normally |
|  | 4 | i s | Free | Free | 7894.641 | 7971.72 | 7911.426 | 0.834148 | 0.1748 | 0.1914 | 9.2% sample in one trajectory |
| **Trouble with taste** | 1 | i s@0 | Free | Free | 8030.424 | 8062.822 | 8037.435 | 0 |  |  |  |
|  | 1 | i s | Free | Same within class | 8036.769 | 8061.068 | 8042.027 | 0 |  |  |  |
|  | 2 | i s | Free | Same within class | |  |  |  |  |  | Model not terminated normally |
|  | **2** | **i@0 s** | **Free** | **Free** | **7872.504** | **7917.051** | **7882.144** | **0.843778** | **0** | **0** |  |
|  | 3 | i@0 s@0 | Free | Free | 7791.63 | 7844.276 | 7803.023 | 0.725816 | 0.3879 | 0.4031 |  |
|  | 3 | i s | Free | Same within class | |  |  |  |  |  | Model not terminated normally |
| **Trouble with coughing** | 1 | i s | Free | Free | 8239.043 | 8279.658 | 8247.924 | 0 |  |  |  |
|  | 1 | i s | Free | Same within class | 8242.831 | 8267.2 | 8248.159 | 0 |  |  |  |
|  | **2** | **i s** | **Free** | **Free** | **8169.999** | **8222.798** | **8181.544** | **0.854385** | **0.0001** | **0.0002** |  |
|  | 2 | i s | Free | Same within class | |  |  |  |  |  | Model not terminated normally |
|  | 3 | i s | Free | Free | 8141.249 | 8206.233 | 8155.458 | 0.753 | 0.2897 | 0.3079 | 10.1% sample in one trajectory |
|  | 3 | i s | Free | Same within class | |  |  |  |  |  | Model not terminated normally |
| **Trouble talking** | **1** | **i s** | **Free** | **trouble_talk15@0** | **7517.929** | **7554.461** | **7525.901** | **0** |  |  |  |
|  | 1 | i s | Free | Same within class | 7576.279 | 7600.634 | 7581.594 | 0 |  |  |  |
|  | 2 | i s | Free | trouble_talk15@0 | 7265.185 | 7313.895 | 7275.814 | 0.952666 | 0.026 | 0.0323 | 10.6% sample in one trajectory |
|  | 2 | i s | Free | Same within class | |  |  |  |  |  | Model not terminated normally |
| **Reflux** | 1 | i s | Free | Free | 8103.681 | 8144.272 | 8112.538 | 0 |  |  |  |
|  | 1 | i s | Free | Same within class | 8099.31 | 8123.665 | 8104.625 | 0 |  |  |  |
|  | **2** | **i s** | **Free** | **Free** | **8041.679** | **8094.447** | **8053.193** | **0.786283** | **0.0039** | **0.0051** |  |
|  | 2 | i s | Free | Same within class | |  |  |  |  |  | Model not terminated normally |
|  | 3 | i s | Free | Free | 7993.268 | 8058.214 | 8007.44 | 0.883158 | 0.314 | 0.3269 |  |
| **Pain** | 1 | i s | Free | Free | 7606.514 | 7647.129 | 7615.395 | 0 |  |  |  |
|  | 1 | i s | Free | Same within class | 7617.99 | 7642.359 | 7623.319 | 0 |  |  |  |
|  | 2 | i s | Free | Free | 7563.076 | 7615.875 | 7574.62 | 0.717642 | 0.269 | 0.2828 |  |
|  | **2** | **i s** | **Free** | **Same within class** | **7425.702** | **7466.317** | **7434.583** | **0.620009** | **0.0001** | **0.0001** |  |
|  | 3 | i s | Free | Free | 7534.627 | 7599.611 | 7548.836 | 0.635761 | 0.0638 | 0.071 | 10.3% sample in one trajectory |
|  | 3 | i s | Free | Same within class | 7380.607 | 7437.467 | 7393.039 | 0.641112 | 0.0289 | 0.0328 | 11.4% sample in one trajectory |
| Models marked in bold are the selected models.  ^a^ EORTC QLQ-OES18: European Organisation for Research and Treatment of Cancer Quality of Life Oesophageal Cancer Module 18 questionnaire  ^b^ AIC: Akaike Information Criterion  ^c^ BIC: Bayesian Information Criterion  ^d^ Sample-size adjusted BIC  ^e^ VLMR: Vuong-Lo-Mendell-Rubin test  ^f^ aLMR: adjusted Lo-Mendell-Rubin test | | | | | | | | | | | |
